# Editorial: Phenylpropanoid metabolism in plants: functional diversity, stress resilience, and biotechnological applications

**DOI:** 10.3389/fpls.2026.1976700

**Published:** 2026-09-02

**Authors:** Vincent Ninkuu, Oluwaseun Olayemi Aluko, Xiupeng Mei, Takayuki Tohge

**Affiliations:** 1 National Nanfan Research Institute, Chinese Academy of Agricultural Sciences, Sanya, China; 2 Key Laboratory of Ministry of Agriculture for Germplasm Resources, Conservation and Utilization of Cassava, Chinese Academy of Tropical Agriculture, Danzhou, China; 3 Maize Research Institute Southwest University, Chongqing, China; 4 Graduate School of Biological Sciences, Nara Institute of Science and Technology (NAIST), Ikoma, Nara, Japan

**Keywords:** environmental cues, metabolic networks, multi-omics applications, phenylpropanoids, plant interactions

**Figure 1:**
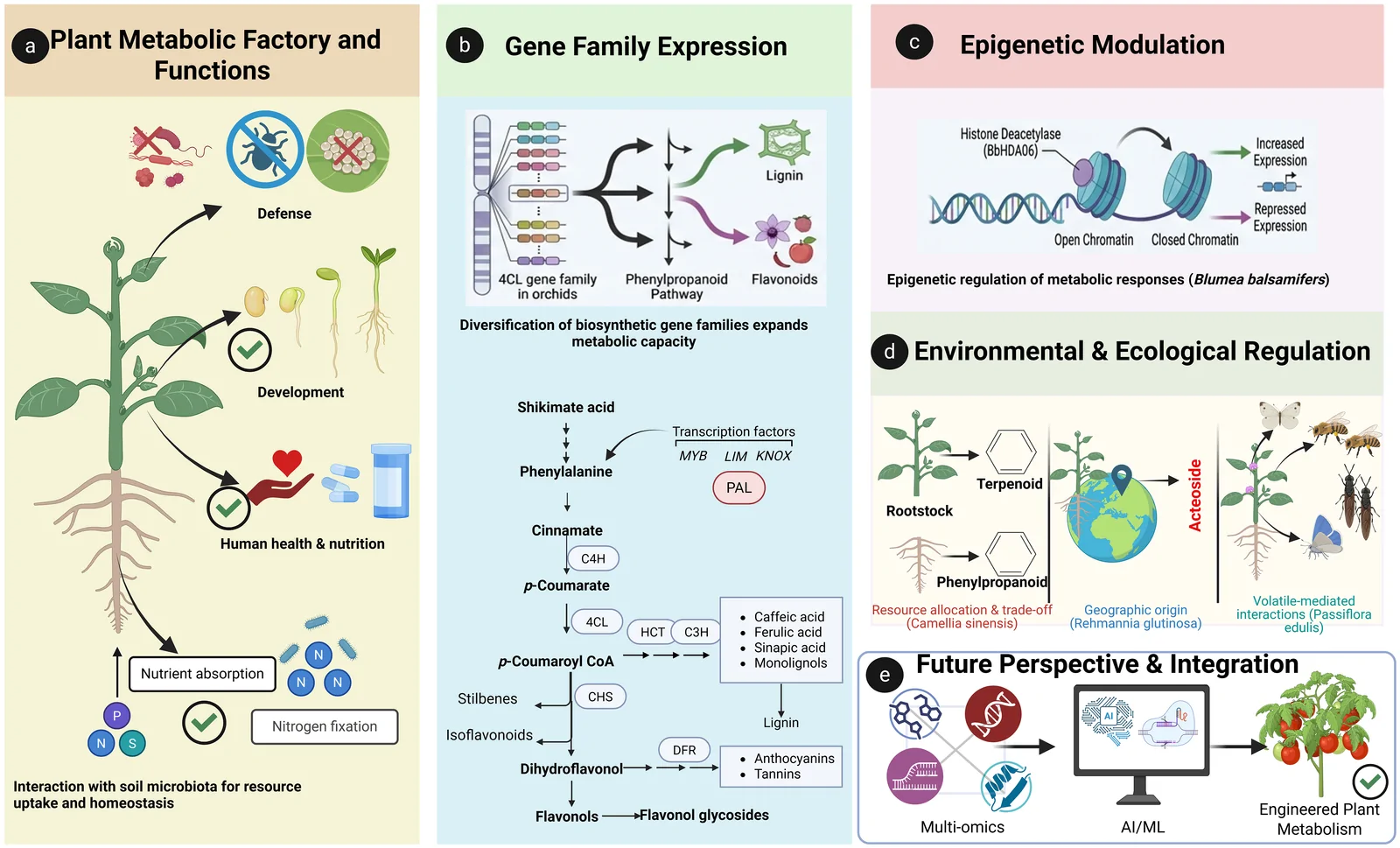
Multilayered regulation of plant phenylpropanoid: genetics, epigenetic, environmental, and ecological interactions. **(a)** Plant metabolic factory and functions **(b)** Gene expression **(c)** Epigenetic modulation **(d)** Environmental and ecological regulation. **(e)** Integration of multi-omics, machine learning, genome editing, and synthetic biology in phenylpropanoid research.

Plants are remarkable metabolic factories, constantly producing a vast array of specialized metabolites that shape their interactions with the environment, defend against biotic and abiotic stresses, facilitate symbiosis, regulate development, and provide essential resources for human health and agriculture (Battat et al., 2019; Ninkuu et al., 2025; Liu et al., 2024; Méteignier et al., 2023; Xiao et al., 2026) (**Figure 1a**). Despite this, plant metabolism continues to evolve novel biochemical strategies through dynamic, context-dependent systems shaped by genetic variation, evolutionary processes, ecological interactions, and environmental conditions (Hanusch et al., 2026; Ninkuu et al., 2022; Tohge et al., 2018). Recent studies compiled in this Research Topic collectively demonstrate that plant chemodiversity is driven by intricate coordination between regulatory networks, metabolic pathways, and environmental signals. These studies, ranging from molecular evolution of biosynthetic enzymes to the ecological consequences of volatile compounds, collectively demonstrate how plants optimize their chemical architecture, highlighting novel perspectives on harnessing metabolic diversity for sustainable agriculture and biotechnological benefits.

One fundamental question in plant metabolism is how the expansion and diversification of gene families drive the evolution of chemical diversity. This question was addressed by Kaur et al. through molecular characterization of the 4-Coumarate CoA ligase (4CL) gene family in orchids. 4-Coumarate CoA ligase is a key enzyme in the phenylpropanoid pathway involved in metabolic flux distribution by catalyzing the conversion of *p*-coumaric acid into *p*-coumaroyl-CoA. This key branching metabolite simultaneously feeds both the lignin and flavonoid pathways, producing a diverse array of metabolites (Ninkuu et al., 2023). Kaur et al. in-silico analysis highlighted the structural diversity, evolutionary conservation, and potential functional specialization of 4CL members in orchids (**Figure 1b**). A comprehensive understanding of the 4CL enzyme can help elucidate the evolution of novel phenylpropanoid metabolites by plants.

Although gene-family diversification expands the metabolic capacity for chemical diversification, this is dynamically regulated by transcriptional, epigenetic, and environmental factors. Hence, plant metabolism is driven by interconnected networks of biosynthetic genes, signaling pathways, transcription factors, and other regulatory mechanisms. Zheng et al. demonstrated this complexity in a transcriptome analysis of leaves from the upper and mid portions of *Xanthoceras sorbifolia*, revealing distinctions in flavonoid production between leaf tissues and emphasizing that flavonoid accumulation is driven by coordinated pathway components rather than by biosynthetic genes alone. The study also noted that higher flavonoid accumulation was associated with enhanced antioxidant capacity and stress tolerance, with *FLS*, *4CL*, and *FG3* regulating this process. These findings demonstrate that chemodiversity is not solely driven by expansion of biosynthetic capacity, but also by context-dependent modulation of metabolic flux.

Luo et al.'s report has further expanded our knowledge of the regulatory landscape of phenylpropanoid metabolism in *Blumea balsamifera*. This study elucidated the epigenetic regulatory role of histone deacetylase *BbHDA6* in flavonoid accumulation under abiotic stress, highlighting an emerging paradigm demonstrating that the metabolic diversity of plants is not merely encoded by DNA sequences, but is rather modulated at the chromatin level (**Figure 1c**). Overexpression of *BbHDA6* enhanced osmotic stress tolerance and modulated salt-stress responses in a developmental stage-dependent manner. Transcriptome profiling of *OE-BbHDA6 Arabidopsis* plants revealed altered expression of key abiotic stress-responsive and flavonoid biosynthetic genes. In *Nicotiana benthamiana*, *OE-BbHDA6* reduced flavonoid contents by approximately 30―37%. This mechanism is therefore an essential layer of metabolic adaptation that enhances plants’ adjustment to their metabolic output under varying environmental conditions.

Elsewhere, Ma et al. highlighted that, in addition to genetic and epigenetic regulatory factors that determine plant metabolic potential, environmental and ecological factors also significantly influence chemical outcomes. Their study showed that rootstock genotypes mediate metabolic trade-offs between terpenoids and phenylpropanoids in *Camellia sinensis* by operating within a resource-allocation framework in which investing in one metabolic class influences the other. This shows that rootstock selection can reshape metabolic allocation and thereby modify quality-related traits without altering the scion genotype. This study specifically noted that hetero-grafting reduced phenylpropanoid (catechins) and caffeine accumulation while enhancing fatty acid and volatile terpene levels by simultaneously downregulating *CsPAL*, *Cs4CL*, and *CsTCS* and upregulating *CsHMGR* and *CsDXS.*

In related findings, Zhang et al. demonstrated that geographical origin can influence acteoside metabolism in *Rehmannia glutinosa* by reprogramming its transcriptome and metabolomic architecture in a coordinated manner (**Figure 1d**). This study showed that environmental cues influence the molecular signature underpinning medicinal plant quality. Specifically, Henan-grown roots showed coordinated upregulation of *PAL*, *C4H*, *4CL*, *TyDC*, and *UGT*, supporting enhanced flux through the phenylpropanoid and tyrosine-derived branches of acteoside biosynthesis. Understanding the environmental factors that influence medicinal plants’ metabolism is essential for optimizing cultivation practices, standardizing these crops, and preserving the authenticity of products derived from them.

Plants’ internal regulations can also impact their ecological functions. For instance, the volatile profile in specific cultivars of *Passiflora edulis* (passion fruit) influences the feeding preference of thrips. According to Li et al., plant metabolites can mediate ecological signaling cascades, enhancing plant-organism interactions by using volatile compounds as communication signals for pollinators, herbivores, and microorganisms. Leaf volatile organic compounds (VOCs) in *P. edulis* influence thrips preference and resistance. A total of 87 differential VOCs, metabolically associated with the phenylpropanoid and α-linolenic acid pathways, were identified, including benzaldehyde and (Z)-3-hexenol as key compounds. Whereas benzaldehyde attracted 58% of thrips, (Z)-3-hexenol repelled 22% of them. This mechanism offers an interesting opportunity for future translational studies to develop environmentally friendly pest management products and provides clues for breeding targets to address pest resistance.

These studies collectively showed that plant phenylpropanoids is generated through an intricate network of regulators, including transcription factors, gene families, environmental conditions, epigenetic modifications, and ecological interactions, rather than through isolated pathways. The intricate interactions among these factors equip plants to respond to external and internal stimuli.

Future research should move beyond descriptive metabolite profiling toward predictive models that connect genotype, regulatory state, environment, and metabolic phenotype. Integrating multi-omics with machine learning, genome editing, and synthetic biology could enable researchers to predict and experimentally redirect metabolic flux under defined environmental conditions (**Figure 1e**). Predicting how environmental conditions, genetic variation, and regulatory mechanisms fine-tune metabolite production provides an opportunity to improve crop breeding, medicinal plant cultivation, and the sustainable production of natural products from plants (Feng et al., 2024).

The contributions in this Research Topic reiterate that metabolites are dynamic outputs of environmental control, evolutionary history, and ecological relationships, rather than mere chemical molecules accumulated in plant tissues. Therefore, harnessing the potential of plant phenylpropanoid requires reframing an integrated approach beyond cataloging metabolites towards elucidating the biological principles governing their emergence, modulation, and functions. Moreover, the recent impact of climate change and growing demand for sustainable resource production have increased attention on deciphering plants’ chemical language as alternative strategies to develop resilient crops, novel bioactive compounds, and build a sustainable future.
